# Substitution of D701N in the PB2 protein could enhance the viral replication and pathogenicity of Eurasian avian-like H1N1 swine influenza viruses

**DOI:** 10.1038/s41426-018-0073-6

**Published:** 2018-05-02

**Authors:** Suli Liu, Wenfei Zhu, Zhaomin Feng, Rongbao Gao, Junfeng Guo, Xiyan Li, Jia Liu, Dayan Wang, Yuelong Shu

**Affiliations:** 1 National Institute for Viral Disease Control and Prevention, Collaboration Innovation Center for Diagnosis and Treatment of Infectious Diseases, Chinese Center for Disease Control and Prevention, Key Laboratory for Medical Virology, National Health and Family Planning Commission, Beijing, 102206 China; 2 School of Public Health (Shenzhen), Sun Yat-sen University, Guangdong, 510275 China

## Abstract

Eurasian avian-like H1N1 (EA H1N1) swine influenza viruses (SIVs) have become predominant in pig populations in China and have recently been reported to have the most potential to raise the next pandemic in humans. The mutation D701N in the PB2 protein, which accounts for 31% of H1N1 SIVs, has previously been shown to contribute to the adaptation of the highly pathogenic H5N1 or H7N7 avian influenza viruses in mammals. However, little is known of the effects of this substitution on the EA H1N1 viruses. Herein, we investigated the contributions of 701N in the PB2 protein to an EA H1N1 SIV (A/Hunan/42443/2015(H1N1), HuN EA-H1N1), which had 701D in the PB2 protein. Our results found that viral polymerase activity, viral replication, and pathogenicity in mice were indeed enhanced due to the introduction of 701N into the PB2 protein, and the increased viral growth was partly mediated by the host factor importin-α7. Thus, substantial attention should be paid to the D701N mutation in pig populations.

## Introduction

Influenza A virus infection usually causes substantial mortality and morbidity. As the genetic mixing vessels for avian and human influenza viruses, pigs are the intermediate hosts for the adaption and pathogenicity of avian influenza viruses and, thus, are potentially capable of initiating pandemics in humans^1^. The EA H1N1 SIVs were first detected in 1979^2^ and have caused several human infections in Europe and Asia since then^3–9^. With the increased binding affinity to human-type receptors and the enhanced transmissibility in mammals, EA H1N1 virus has become one of the candidates with the greatest likelihood to raise pandemics^1,10,11^.

The viral polymerase is a major determinant of interspecies transmission and pathogenesis^12–15^. The D701N mutation in the PB2 protein was originally observed upon the adaption of H3N2, H5N1, and H7N7 viruses in mice^16–18^ and was associated with enhanced viral polymerase activity and pathogenicity^16–20^. Increased interaction between 701N and cellular importin-α, a component of the nuclear import machinery, was believed to contribute to adaptation, resulting in enhanced nuclear import of vRNPs^21,22^.

In public databases, only D is found at position 701 in the PB2 protein of avian H1N1 influenza viruses, while both D and N are observed in swine and human H1N1 influenza viruses, with the percentages of N being 31.175 and 0.126%, respectively (Supplementary Table 1). With the prevalence of EA H1N1 viruses in pigs, various reassortant patterns among EA H1N1 viruses and 2009pdmH1N1 or other co-circulated swine influenza viruses have been detected^1,23–25^. One of the newly emerged reassortants, with two surface genes from the EA H1N1 virus and six internal genes from the 2009pdmH1N1 and classical swine influenza viruses, was reported to have higher pathogenicity in mice compared with the un-reassorted EA H1N1 virus^9^. These viruses contained amino acid 701D in the PB2 protein. Considering the possibility of EA H1N1 SIVs causing a future human influenza pandemic and the great significance of the 701N mutation in host adaptation, our study focused on the effects of the substitution of the mammalian signature 701N in the PB2 protein on this newly emerged reassortant (A/Hunan/42443/2015(H1N1), HuN EA-H1N1, accession nos. EPI691392-EPI691399 in the GISAID database) isolated from a severely ill child. This virus was a genetic reassortant of the EA H1N1 virus and had a D at position 701 in the PB2 protein^9^.

## Results

### D701N increased the polymerase activity of the EA H1N1 virus in human cells

**Fig. 1:**
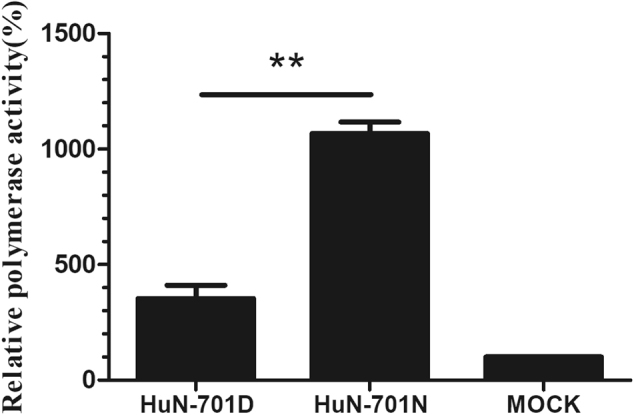
Polymerase activity of the PB2 mutants in human cells. 293T cells were transfected with a pFluc plasmid expressing negative-sense virus-like RNA and encoding firefly luciferase (Fluc) and a pRluc plasmid expressing the Renilla luciferase gene (Rluc) as an internal control (Promega). The 293T cells were also co-transfected with plasmids expressing the wild-type (701D) and mutated (701 N) PB2, PB1, NP, and PA segments derived from the A/Hunan/42443/2015(H1N1) viruses. After being cultured at 37 °C for 24 h, cell lysates were used to measure the Fluc and Rluc activity levels. Mock transfection with the two reporter constructs only was set to 100%. The means ± SD of triplicate experiments are shown. **P < 0.01

To study the effects of the D701N mutation on the viral polymerase activity of the EA H1N1 virus, we reconstituted viral RNPs in 293T cells. Mutation D701N was shown to increase polymerase activity significantly (*P* < 0.01), with a 3-fold increase (Fig. 1).

### D701N enhanced viral morbidity and mortality in mice

**Fig. 2:**
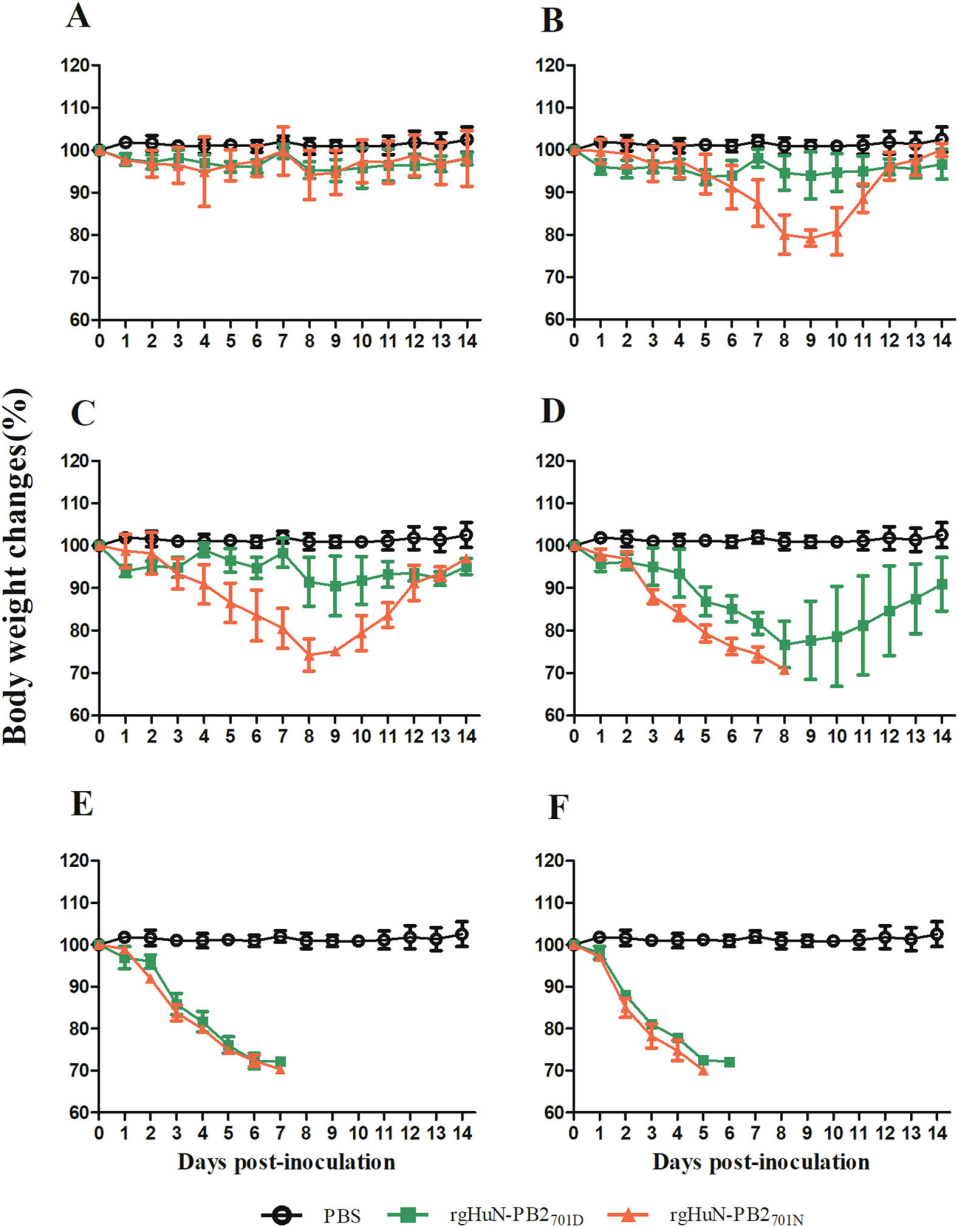
Infection of C57BL/6 mice with recombinant HuN EA-H1N1 viruses. Eight- to ten-week-old female C57BL/6 mice (*n* = 5/group) were inoculated intranasally with 10^1^ (**a**), 10^2^ (**b**), 10^3^ (**c**), 10^4^ (**d**), 10^5^ (**e**), and 10^6^ TCID_50_ (**f**) of the recombinant viruses. Mice receiving PBS were used as controls. Weight loss was monitored for 14 days

**Fig. 3:**
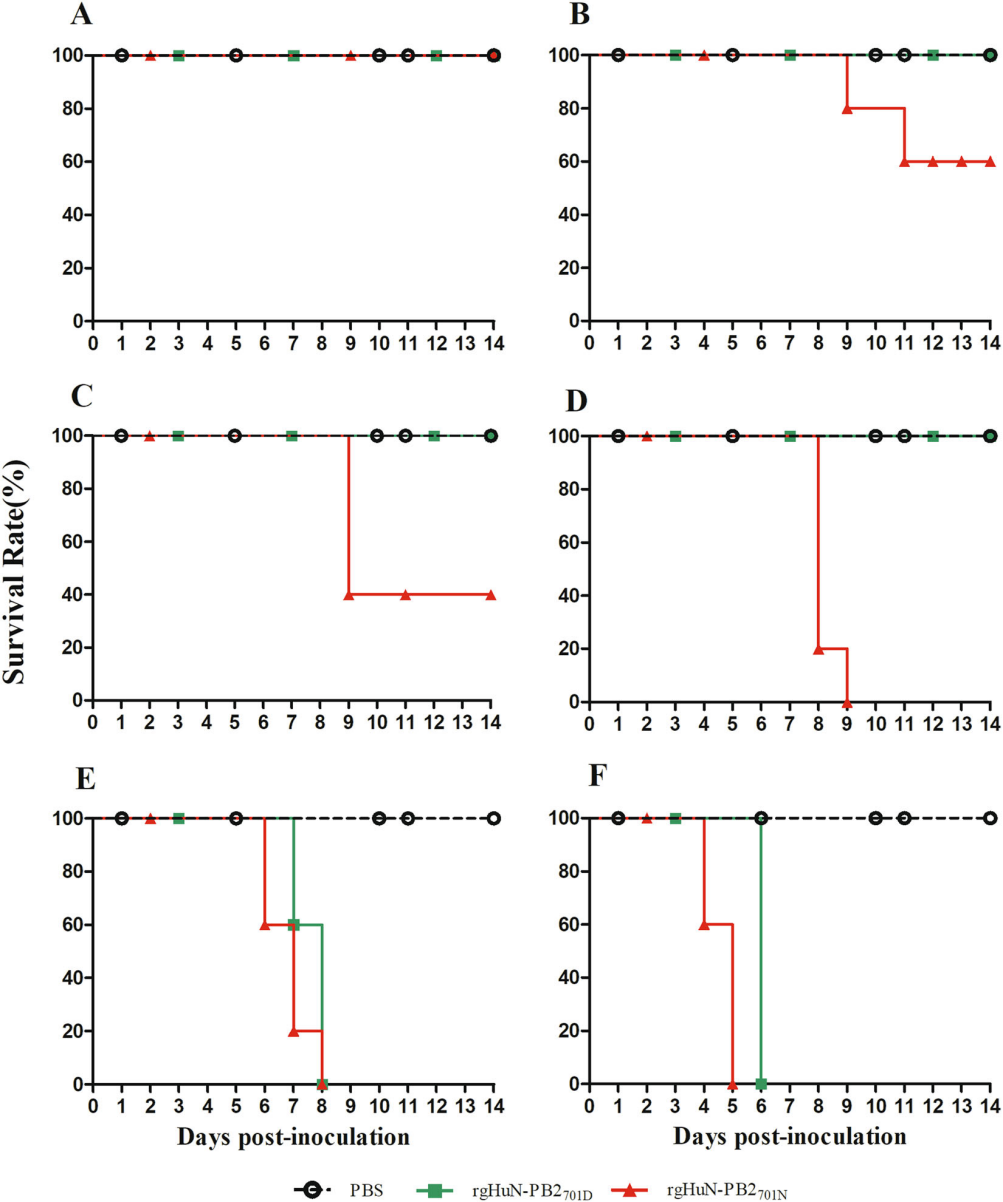
Mortality of the recombinant HuN EA-H1N1 viruses in mice. Eight- to ten-week-old female C57BL/6 mice (*n* = 5/group) were inoculated intranasally with 10^1^ (**a**), 10^2^ (**b**), 10^3^ (**c**), 10^4^ (**d**), 10^5^ (**e**), 10^6^ TCID_50_ (**f**) of the recombinant viruses. Mice receiving PBS were used as controls. Survival was monitored for 14 days

To evaluate the effects of the D701N mutation on pathogenicity in mammals, 10-fold serially diluted recombinant viruses (rgHuN-PB2_701D_ and rgHuN-PB2_701N_) were inoculated into groups of five mice. At the inoculation dose of 10^1^ TCID_50_, none of the mice died, and the body weight loss was <10% for both viruses (Figs. 2a and 3a). At the dose of 10^2^ TCID_50_, two mice inoculated with the rgHuN-PB2_701N_ viruses died at 9 dpi and 11 dpi, respectively, and the peak body weight loss at 9 dpi was >20% vs. <10% for mice inoculated with the rgHuN-PB2_701D_ viruses (Figs. 2b and 3b). At the dose of 10^3^ TCID_50_, three mice inoculated with the rgHuN-PB2_701N_ viruses died at 9 dpi, but no rgHuN-PB2_701D_-inoculated mice died, and the body weight loss was <10% (Figs. 2c and 3c). At the dose of 10^4^ TCID_50_, all five mice inoculated with the rgHuN-PB2_701N_ viruses died within 9 dpi, but no rgHuN-PB2_701D_-inoculated mice died, and the body weight loss was >20% (Figs. 2d and 3d). At the doses of 10^5^ TCID_50_ and 10^6^ TCID_50_, significant body weight loss was observed in both groups of mice, but the rgHuN-PB2_701N_-inoculated mice died earlier (Figs. 2e, f and 3e, f). The MLD_50_ values of the rgHuN-PB2_701D_ and rgHuN-PB2_701N_ viruses were ≥10^4.5^ and 10^2.5^ TCID_50_ (Fig. 3), while the MID_50_ were 10^1.5^ and 10^0.5^ TCID_50_, respectively (Table 1). These results indicated that the introduction of the 701N mutation into the PB2 protein of A/Hunan/42443/2015(H1N1) increased the viral virulence in mice.

**Table 1:** Seroconversions of mice inoculated with recombinant viruses^a^

Virus and dose, log_10_ TCID_50_/50 μl	Hemagglutination inhibition titer^b^ (HuN EA-H1N1 virus antigen)					MID_50_, log_10_ TCID_50_^c^
	Mouse 1	Mouse 2	Mouse 3	Mouse 4	Mouse 5	
rgHuN-PB2_701D_						1.5
6	ND	ND	ND	ND	ND	
5	ND	ND	ND	ND	ND	
4	640	640	640	640	640	
3	320	320	640	640	640	
2	20	≤10	640	320	≤10	
1	<10	40	<10	160	320	
rgHuN-PB2_701N_						0.5
6	ND	ND	ND	ND	ND	
5	ND	ND	ND	ND	ND	
4	ND	ND	ND	ND	ND	
3	640	640	ND	ND	ND	
2	640	640	640	ND	ND	
1	640	160	40	320	320	
PBS	<10	<10	<10	<10	<10	

MID_50_ 50% mouse infectious dose, ND not determined due to death of mice (treated as positive), TCID_50_ 50% tissue culture infectious dose

^a^Mice serum was collected at 14 days post inoculation

^b^An HI titer of <40 was recognized as negative

^c^MID_50_ was determined by the Spearman–Karber method^32^; TCID_50_ was calculated using the Reed-Muench formula^33^

### D701N enhanced viral pathogenicity in mice

**Fig. 4:**
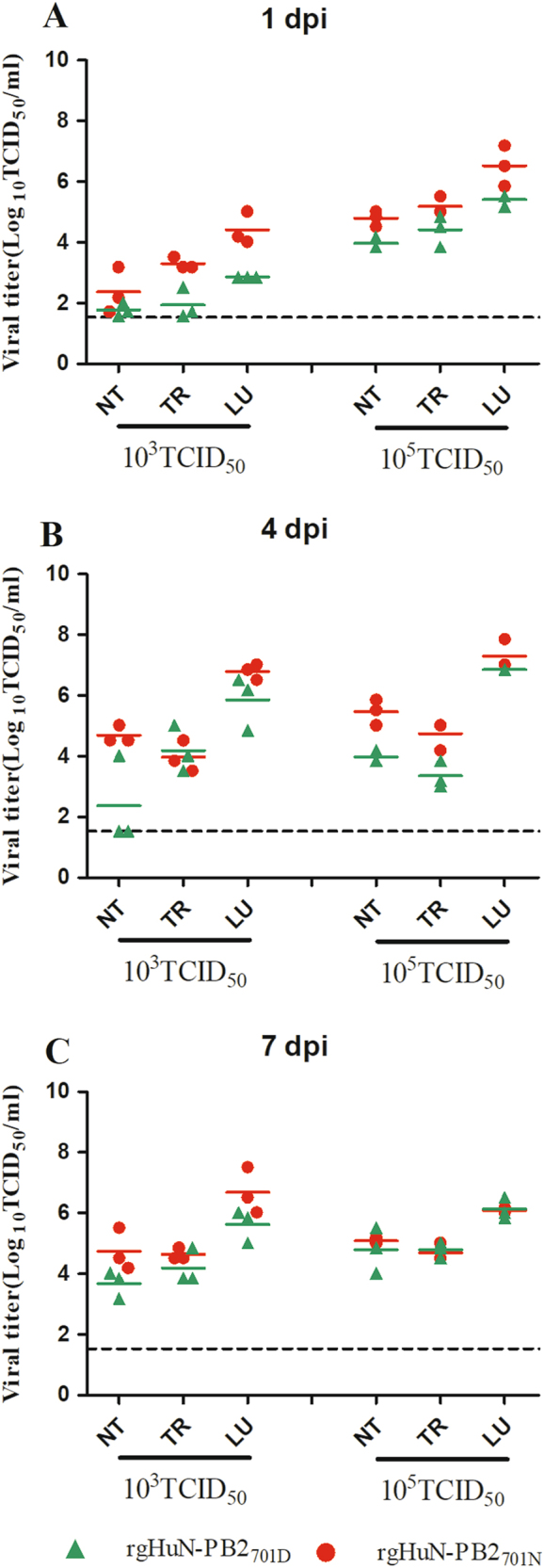
Replication of the recombinant viruses in the respiratory tracts of mice. Eight- to ten-week-old female C57BL/6 mice (*n* = 3/time-point) were inoculated intranasally with 10^3^ TCID_50_ (left) or 10^5^ TCID_50_ (right) of the recombinant viruses. Animals were euthanized at 1 (**a**), 4 (**b**), and 7 (**c**) dpi. Viral titers in the tissues were determined on MDCK cells. NT Nasal turbinate, TR trachea, LU lung

**Fig. 5:**
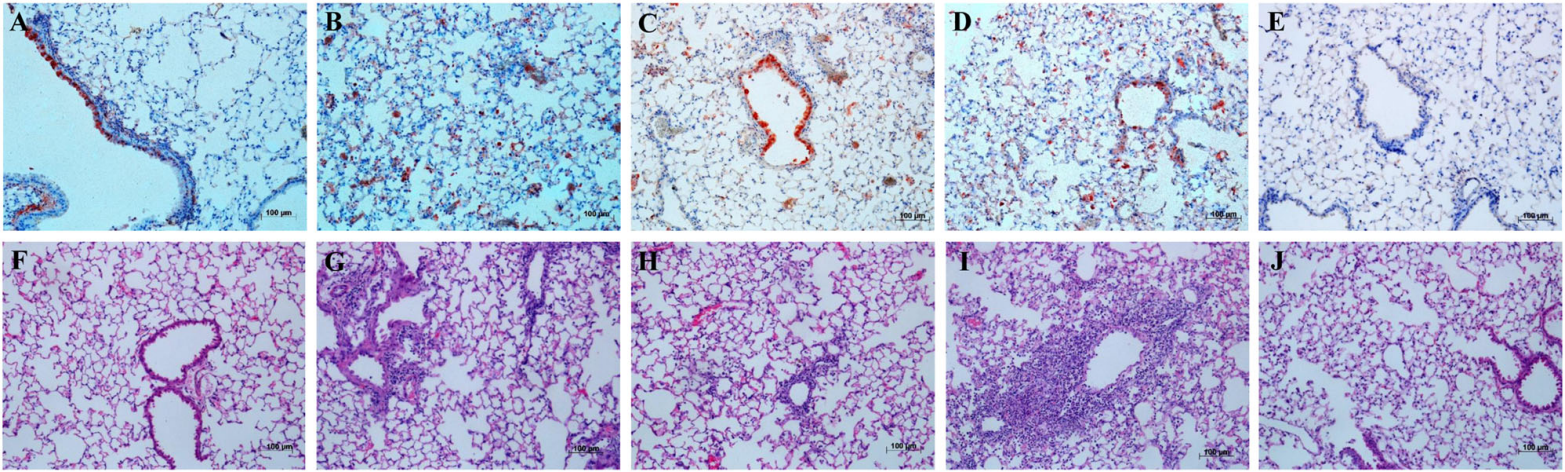
Replication and pathological changes in the lungs of mice inoculated with recombinant viruses. Eight- to ten-week-old female C57BL/6 mice (*n* = 3/time-point) were inoculated intranasally with 10^3^ TCID_50_ of the rgHuN-PB2_701D_ viruses (**a**, **f**) and rgHuN-PB2_701N_ viruses (**c**, **h**) or 10^5^ TCID_50_ of the rgHuN-PB2_701D_ viruses (**b**, **g**) and rgHuN-PB2_701N_ viruses (**d**, **i**). Mice receiving PBS (**e**, **j**) were used as controls. Mice were killed at 4 dpi, and the lung lobes were used for NP antigen staining (upper panel) and hematoxylin and eosin (**H**&**E**) staining (lower panel)

Replication of the HuN EA-H1N1 recombinant viruses was detected in the nasal turbinates, tracheas and lungs of C57BL/6 mice, with the highest viral titers in the lungs. Viral titers in all tissues of mice inoculated with the rgHuN-PB2_701N_ viruses were higher than those of mice inoculated with the rgHuN-PB2_701D_ viruses, except for the mice inoculated with viruses at the dose of 10^5^ TCID_50_ at 7 dpi (Fig. 4). Lung viral titers also correlated with the level of pathogenicity in the lungs, causing overt peribronchiolitis and pulmonary alveolitis (Fig. 5). At the dose of 10^3^ TCID_50_, virus replication was largely restricted to the bronchial epithelium, resulting in epithelial cell exfoliation and mononuclear infiltration (Fig. 5a, c, f, h), in contrast to the dose of 10^5^ TCID_50_, where virus particles were predominantly detected in alveolar cells, leading to severe alveolar destruction (Fig. 5b, d, g, i). Inflammation and virus replication in the lungs were more prominent in mice inoculated with the rgHuN-PB2_701N_ viruses than in mice inoculated with the rgHuN-PB2_701D_ viruses.

### Importin-α7 correlated with the increased growth property of the D701N mutants

**Fig. 6:**
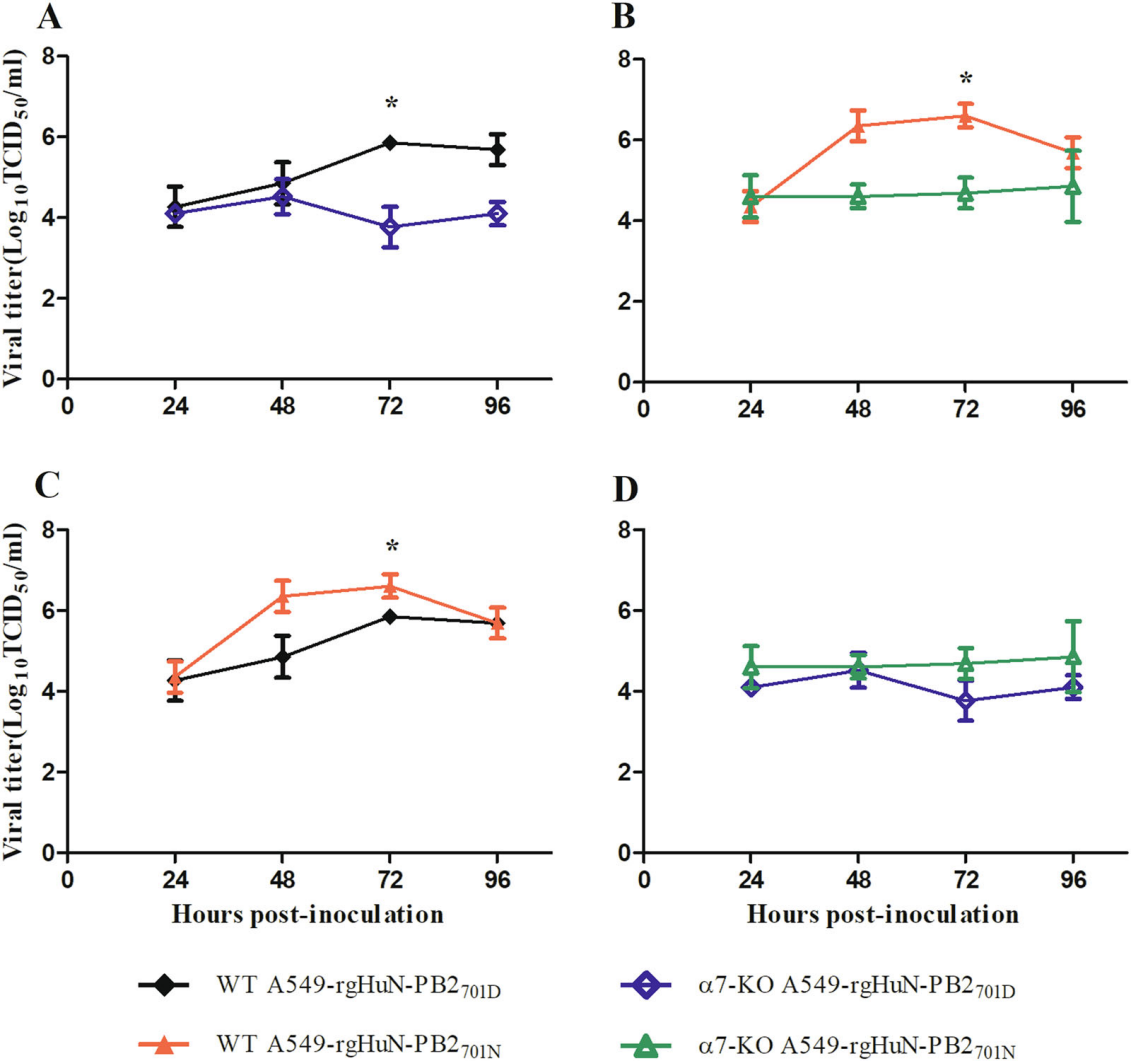
Replication kinetics of the recombinant viruses in wild-type (WT) and importin-α7 knockout (α7-KO) A549 cells. Cells were inoculated with the recombinant viruses at 37 °C, and culture supernatants were harvested at 0, 24, 48, 72 and 96 h post-inoculation (hpi). Virus titers were determined on MDCK cells. **a** Growth curves of the rgHuN-PB2_701D_ viruses in WT (black, filled diamonds) and α7-KO (blue diamonds) A549 cells; **b** Growth curves of the rgHuN-PB2_701N_ viruses in WT (red, filled triangles) and α7-KO (green triangles) A549 cells; **c** Growth curves of the rgHuN-PB2_701D_ viruses (black, filled diamonds) and the rgHuN-PB2_701N_ viruses (red, filled triangles) in WT A549 cells; **d** Growth curves of the rgHuN-PB2_701D_ viruses (blue diamonds) and the rgHuN-PB2_701N_ viruses (green triangles) in α7-KO A549 cells. The results are presented as the means ± SD of three repeated experiments. *P *<* 0.05

To assess whether the enhanced virus replication and pathogenicity by the introduction of the mammalian signature N at position 701 of the PB2 protein of the rgHuN-PB2_701N_ viruses were related to importin-α7, we established an importin-α7 knockout A549 cell line using CRISPR/Cas9 technology and infected the cells with the HuN EA-H1N1 recombinant viruses. As shown in Fig. 6a, b, both rgHuN-PB2_701D_ and rgHuN-PB2_701N_ viruses replicated significantly less at 72 hpi in importin-α7 knockout cells (up to 100-fold less, *P* < 0.05), indicating that importin-α7 was required for the efficient replication of both viruses. In wild-type A549 cells, the rgHuN-PB2_701N_ viruses exhibited significantly higher replication levels than the rgHuN-PB2_701D_ viruses at 72 hpi (up to 6-fold higher, *P* < 0.05); in contrast, in importin-α7 knockout cells, growth of these two viruses had no significant difference, despite the lower replication level of the rgHuN-PB2_701D_ viruses (Fig. 6c, d). These findings suggested that the increased replication of the rgHuN-PB2_701N_ viruses compared with the rgHuN-PB2_701D_ viruses was partly mediated by importin-α7.

### D701N promoted the nuclear import of vRNPs in an importin-α7-dependent manner

**Fig. 7:**
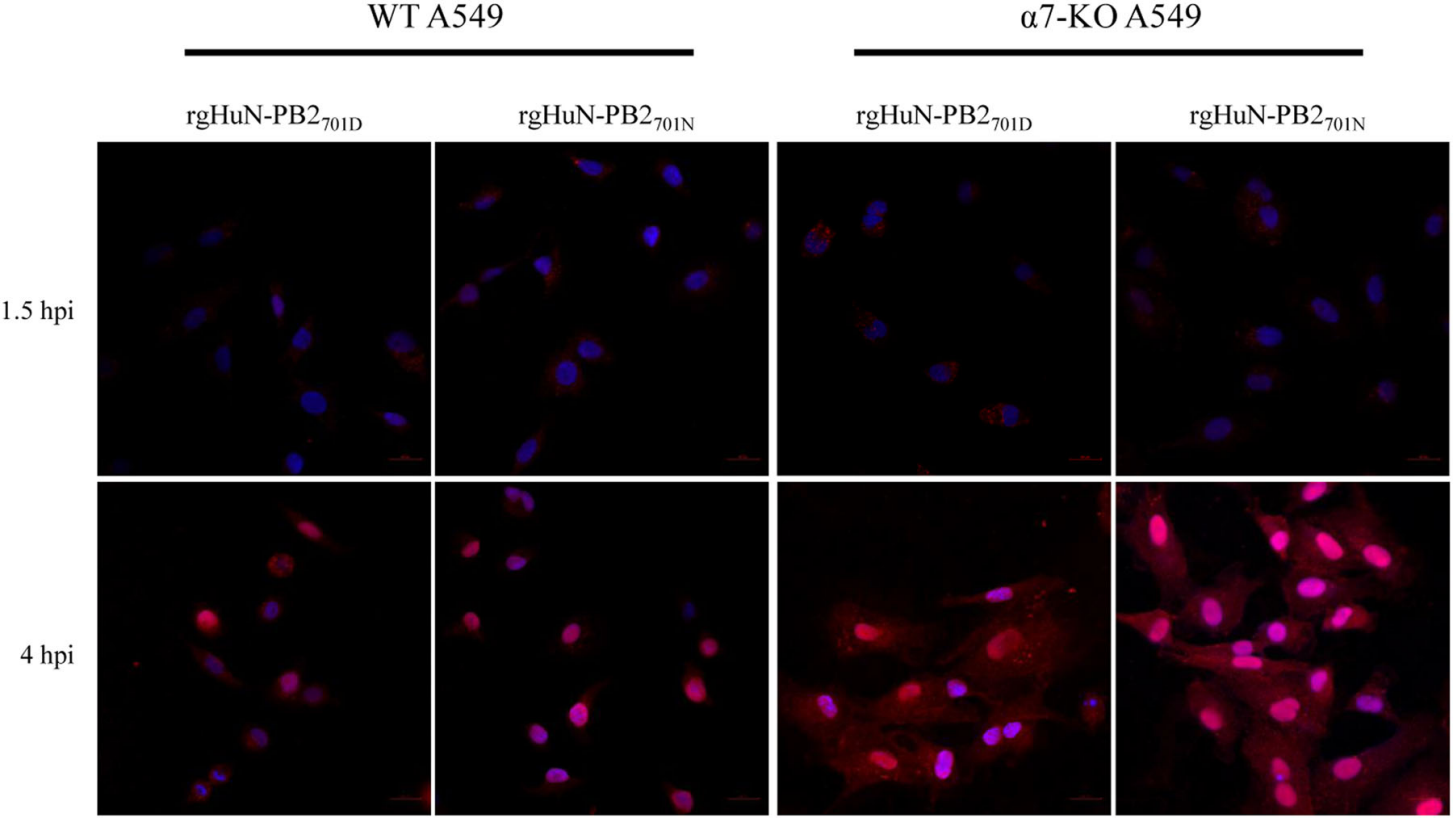
Sub-localization of vRNPs in wild-type (WT) and importin-α7 knockout (α7-KO) A549 cells infected with the recombinant viruses. WT and α7-KO A549 cells were infected with recombinant viruses at 37 °C and were fixed at indicated time points (1.5 hpi early in the life cycle and 4 hpi in the middle of the life cycle). Cells were then immunostained with an antibody against NP and an Alexa Fluor 594-conjugated secondary antibody. Cell nuclei were stained with DAPI

To further cast light onto the molecular basis of the enhanced viral growth mediated by importin-α7, we infected wild-type and importin-α7 knockout A549 cells with the HuN EA-H1N1 recombinant viruses and detected the subcellular localization of vRNPs by confocal microscopy. At 1.5 hpi, NP proteins formed a nuclear peripheral staining pattern in the cytoplasm (Fig. 7, upper panel), indicating consistent virus entry. At 4 hpi, nearly all NP proteins in wild-type A549 cells had accumulated in the cell nucleus, while nuclear accumulation of NP was reduced in importin-α7 knockout A549 cells (Fig. 7, lower panel), suggesting that importin-α7 played a role in the nuclear import of vRNPs. In wild-type A549 cells, the percentage of positive cells induced by the rgHuN-PB2_701N_ viruses was obviously higher than that induced by the rgHuN-PB2_701D_ viruses (Fig. 7, lower panel, left), while in importin-α7 knockout A549 cells, the difference in nuclear import of vRNPs between these two viruses was not obvious (Fig. 7, lower panel, right). These results were consistent with the findings on the growth properties (Fig. 6) and indicated that importin-α7 might be associated with the enhanced nuclear import of vRNPs of EA H1N1 SIVs with a D701N substitution.

## Discussion

After a long evolutionary term, EA H1N1 SIVs are now predominant in pig populations in China^1^. Frequent reassortments among EA H1N1 viruses and other co-circulated SIVs have occurred, and EA H1N1 viruses with different gene cassettes exhibit different phenotypes in mammal models^1,9,23–25^. Some of them have been documented to cause human infections, with varied clinical manifestations^5,6,8,9^. Sequence analysis in public databases has found that approximately one-third of the swine H1N1 influenza viruses have an N at position 701 in the PB2 protein (Supplementary Table 1), which has been reported to contribute to the enhanced replication and pathogenicity of the highly pathogenic H5N1, H7N7, and H7N9 avian influenza viruses in mammals^17–20^. In this study, we demonstrated that introduction of the 701N mutation into the PB2 protein of EA H1N1 SIVs enhanced viral replication and pathogenicity in mice. Thus, substantial attention should be paid when evaluating the biological properties and biosafety of EA H1N1 SIVs.

Adaptation mediated by D701N involved a switch in the usage of importin-α isoforms. Viruses with the avian signature 701D in PB2 preferred importin-α1 and importin-α3, while viruses with the mammalian signature 701N primarily depended on importin-α1 and importin-α7 for optimal viral growth in mammalian cells^22,26^. Our viral growth curve showed that the growth of both recombinant viruses relied on importin-α7, but the higher growth level of the rgHuN-PB2_701N_ viruses compared with the rgHuN-PB2_701D_ viruses also required importin-α7 (Fig. 6), likely due to the differential nuclear import efficiencies of vRNPs (Fig. 7). Moreover, previous studies demonstrated that importin-α7 was crucial for enhanced viral replication and severe damage in mice using importin-α7 knockout mice^27–29^. Therefore, future studies will be required to investigate the molecular mechanism of the enhanced viral replication and pathogenicity mediated by the D701N mutation using the importin-α7 knockout mouse model.

In summary, our findings provided the first insights into the effects of the D701N mutation in the PB2 protein of EA H1N1 SIVs on viral replication and pathogenicity and further investigated the importin-α7 dependency of the increased viral growth resulting from the D701N mutation.

## Materials and methods

### Cell culture

Madin-Darby canine kidney (MDCK) cells, human embryonic kidney (293T) cells and human type II alveolar epithelial (A549) cells were grown and maintained in Dulbecco’s modified Eagle’s medium (DMEM; Invitrogen) supplemented with 10% fetal bovine serum (FBS; Invitrogen), HEPES (10 Mm; Invitrogen), penicillin (100 units/ml), and streptomycin (100 μg/ml; Invitrogen).

Importin-α7-KO A549 cells were generated using CRISPR/Cas9 technology as described as previously^30^. In brief, two pairs of small guide RNAs (sgRNAs) targeting exons 5 and 6 of the human importin-α7 gene were designed and cloned into the pX459 vector, and the recombinant plasmids were then co-transfected into A549 cells to perform genome modification. Successful knockout of importin-α7 in A549 cells was confirmed by DNA sequencing and Western blot analysis. For the detection of importin-α7, a rabbit anti-importin-α7 antibody (Abcam) was used, followed by a horseradish peroxidase-conjugated goat anti-rabbit secondary antibody (Zhongshanjinqiao Biotech). Tubulin (Abcam) was used as a loading control.

### Site-directed mutagenesis

All eight gene segments of influenza virus A/Hunan/42443/2015(H1N1) were amplified by RT-PCR and cloned into the pHW2000 vector. A plasmid with a single point mutation at residue 701 in the PB2 gene was generated with the following primers: PB2 D701N Forward, GGG CAA AGA A**A**A CAA GAG; PB2 D701N Reverse, CTC TTG T**T**T TCT TTG CCC. The presence of the introduced mutations and the absence of additional unwanted mutations were verified by sequencing of the whole cDNA.

### Generation of recombinant viruses and virus titration

The wild-type HuN EA-H1N1 virus encoded a 701D in the PB2 protein. The recombinant viruses (rgHuN-PB2_701D_ and rgHuN-PB2_701N_) were generated by reverse genetics, as described previously^31^. In general, the eight gene segments were cloned into the pHW2000 vector and were co-transfected into 293T/MDCK co-cultured monolayers for 24 h. Culture supernatants were propagated in 9–11-day embryonated chicken eggs. Virus stocks were sequenced for verification, and virus titrations were determined on MDCK cells.

### Polymerase activity assay

Around 293 T cells were co-transfected with pHW2000-PB2/PB1/PA/NP (0.5 μg each) together with the reporter constructs (0.5 μg each) pFluc (encoding firefly luciferase flanked by the noncoding regions of the influenza NP segment to produce an artificial influenza NP-like RNA segment) and pRluc (pRL-TK, encoding *Renilla* luciferase for normalization, Promega) to reconstitute vRNPs. Mock transfections were performed with the two reporter constructs only. Luciferase activity was measured after 24 h of incubation according to the Promega Dual-Luciferase Reporter Assay kit protocol.

### Animal experiments

Our animal experiments were performed according to the guidelines of the Ethics Committee of the National Institute for Viral Disease Control and Prevention, China CDC (20160226008). Eight- to ten-week-old female C57BL/6 mice (Vital River Laboratories) were anesthetized with 1% pentobarbital sodium and inoculated intranasally with 50 μl of 10-fold serial dilutions of viruses in PBS. Mice receiving PBS were used as controls. Weight loss and survival were observed for 14 days. Mice with weight loss >30% were humanely killed. For detection of virus replication and lung tropism, mice infected with 10^3^ and 10^5^ TCID_50_ of viruses were sacrificed on days 1, 4, and 7 post-infection. The right lung lobes were perfused with 10% neutral buffered formalin for immunohistochemical analysis, and the remaining lung tissues were homogenized for virus titration on MDCK cells.

### Histological analysis

The processed lung lobes were embedded in paraffin and cut into 3–4-μm sections. One section was stained with hematoxylin and eosin to check the pathological changes, and another was immunohistochemically stained against influenza virus antigen with mouse anti-NP, kindly provided by Professor Ningshao Xia, the National Institute of Diagnostics and Vaccine Development for Infectious Diseases, Xiamen University. Antigens of influenza viruses were detected using a peroxidase-conjugated anti-mouse/rabbit secondary antibody (Zhongshanjinqiao Biotech).

### Growth curves

A549 cells were infected at a MOI of 0.1 with the recombinant viruses for multicycle replication under the condition of 5% CO_2_ and 37 °C. After 1 h of incubation, cells were washed twice with PBS, and infection medium containing TPCK-trypsin was added. At time points 0, 24, 48, 72 and 96 h post-infection (hpi), supernatants were collected, and viral titers were determined on MDCK cells.

### Detection of vRNP localization in infected cells by confocal microscopy

Wild-type and importin-α7 knockout A549 cells infected with the recombinant viruses were fixed in 4% paraformaldehyde at 1.5 and 4 hpi. After three washes with PBS, cells were then permeabilized with 0.1% Triton X-100 for 10 min at room temperature. Cells were rinsed once with PBS and then incubated with 2% BSA (blocking reagent) for 30 min at room temperature. Cells were then incubated with mouse anti-NP primary antibody (Abcam) diluted in 2% BSA (1:200) for 60 min at room temperature. After three washes with PBS, cells were incubated with anti-mouse IgG (H+L)/Alexa Fluor 594-conjugated secondary antibody (1:500, Thermo Fisher) for 60 min at room temperature. Cells were washed once with PBS and stained with DAPI (Beyotime Biotechnology) for 10 min at room temperature. Cells were then washed with PBS three times and examined for fluorescence using a Zeiss LSM confocal microscope.

### Statistical analysis

Student’s *t* test (unpaired, two-tailed) was calculated using GraphPad Prism software (GraphPad Software Inc., CA, USA). A *P* value of <0.05 was considered significant.

## Electronic supplementary material

Supplementary Table S1. Summary of amino acid at position 701 in the PB2 protein of H1N1 influenza viruses isolated in Asian countries from 2008 to 2017
