# Supplementary material for: Substitution of D701N in the PB2 protein could enhance the viral replication and pathogenicity of Eurasian avian-like H1N1 swine influenza viruses
Source: Emerg Microbes Infect. 2018 May 2;7:75. doi: 10.1038/s41426-018-0073-6 (PMC5931605; doi:10.1038/s41426-018-0073-6)
Supplement: Supplementary file 1 — Supplementary Table S1. Summary of amino acid at position 701 in the PB2 protein of H1N1 influenza viruses isolated in Asian countries from 2008 to 2017 [file 41426_2018_73_MOESM1_ESM.docx]

Supplementary Table 1. Summary of amino acid at position 701 in the PB2 protein of H1N1 influenza viruses isolated in Asian countries from 2008 to 2017^*^

| Species (PB2 sequence counts) | Percentage of amino acid at position 701 in PB2 (%) | |
| --- | --- | --- |
|  | D | N |
| Avian (36) | 100.000 | 0.000 |
| Swine (674) | 68.843 | 31.157 |
| Human (3970) | 99.849 | 0.126 |

^*^ PB2 protein sequences were downloaded from the public database of Global Initiative on Sharing All Influenza Data (http://platform.gisaid.org). D:Asp, N:Asn.
